# Insecticide resistance in malaria vector mosquitoes at four localities in Ghana, West Africa

**DOI:** 10.1186/1756-3305-4-107

**Published:** 2011-06-16

**Authors:** Richard H Hunt, Godwin Fuseini, Steve Knowles, Joseph Stiles-Ocran, Rolf Verster, Maria L Kaiser, Kwang Shik Choi, Lizette L Koekemoer, Maureen Coetzee

**Affiliations:** 1 Malaria Entomology Research Unit, School of Pathology, Faculty of Health Sciences, University of the Witwatersrand, Johannesburg, South Africa; 2 Vector Control Reference Unit, National Institute for Communicable Diseases of the National Health Laboratory Service, Johannesburg, South Africa; 3 Newmont Mines, Ahafo, Ghana; 4 Malaria Control Programme, AngloGold/Ashanti, Obuasi, Ghana; 5 Gold Fields Limited, Sandown, Johannesburg

## Abstract

**Background:**

Malaria vector control programmes that rely on insecticide-based interventions such as indoor house spraying with residual insecticides or insecticide treated bed nets, need to base their decision-making process on sound baseline data. More and more commercial entities in Africa, such as mining companies, are realising the value to staff productivity of controlling malaria transmission in their areas of operation.

This paper presents baseline entomological data obtained during surveys conducted for four mining operations in Ghana, West Africa.

**Results:**

The vast majority of the samples were identified as *Anopheles gambiae *S form with only a few M form specimens being identified from Tarkwa. *Plasmodium falciparum *infection rates ranged from 4.5 to 8.6% in *An. gambiae *and 1.81 to 8.06% in *An. funestus*. High survival rates on standard WHO bioassay tests were recorded for all insecticide classes except the organophosphates that showed reasonable mortality at all locations (i.e. > 90%). The West African *kdr *mutation was detected and showed high frequencies in all populations.

**Conclusions:**

The data highlight the complexity of the situation prevailing in southern Ghana and the challenges facing the malaria vector control programmes in this region. Vector control programmes in Ghana need to carefully consider the resistance profiles of the local mosquito populations in order to base their resistance management strategies on sound scientific data.

## Background

Malaria remains today the biggest killer of children in Africa [1] and it demands increased attention from control authorities in affected countries. Multi-national corporations operating in Africa recognize the burden malaria places on their staff and its impact on their commercial operations [2,3]. There is an increasing move by these multi-nationals to control malaria within the boundaries of their activities, and in many cases, extending this control to the surrounding communities [4,5].

The traditional methods of protecting work-forces using fogging, prophylaxis, repellents and handing out insecticide treated bed nets to workers (ITNs), have clearly not resulted in the desired outcome and companies are now implementing malaria control through the use of indoor residual house spraying [2,3]. Key to the success of these measures is knowledge of the local vector populations, the species identity, role in transmission and susceptibility to the four classes of insecticides approved by the World Health Organization for use in vector control. Baseline surveys to collect this information need to be carried out prior to implementation of malaria vector control interventions and on-going susceptibility surveys done to guard against the development of increasing insecticide resistance. This is becoming more important as insecticide resistance increases and spreads across Africa.

This paper reports on four such baseline surveys carried out at mining operations in Ghana, West Africa.

## Materials and methods

### Study sites

**Figure 1:**
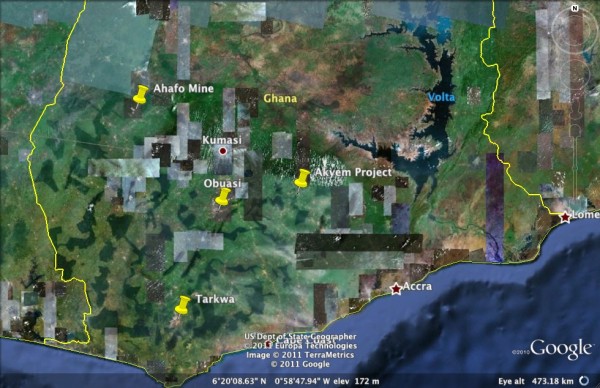
**Google Earth map showing the localities of the four mining sites surveyed in this study**.

Ghana has a relatively high rainfall, situated between 5° and 12° North of the Equator with typical tropical temperatures. The vegetation at the sites sampled was dense degraded forest, mixed with agricultural land. Formal cultivation of cocoa and oil palm is interspersed with secondary forest and subsistence farming with cassava, maize, pineapples, etc. The following gold mining sites (Figure 1) were surveyed for a maximum of two weeks each between 2006-2010:

Obuasi: (6°11'36"N, 1°39'29"W), a town of approximately 150,000 people with local inhabitants living in and around the mining operations. Gold has been mined at Obuasi for over 100 years, much of it open-cast mining. Originally owned by Ashanti Gold Mines, the mine is now operated by AngloGold/Ashanti who have instituted an extensive malaria control programme in the area for the past four years. The mosquitoes used in this survey were collected outside but adjacent to the Obuasi malaria vector control area in July 2010.

Tarkwa/Damang: (5°18'N, 1°59'32"W) Two new mines in this area operated by AngloGold/Ashanti and Randgold Resources were sampled in May 2007 and March 2009.

Ahafo: (7°1'33"N, 2°20'27"W), a Newmont Mines operation, was sampled in December 2006 and June 2008.

Akyem: (6°20'24"N, 1°0'28"W) also operated by Newmont Mines, was sampled in June 2006.

### Mosquito collections

Local villages on the outskirts of the mining operations were visited and permission sought from the chief or local householders to search their houses for indoor resting mosquitoes. Houses were searched at random and those found to yield good numbers of mosquitoes were revisited repeatedly to ensure sufficient samples for the susceptibility tests. The collections were carried out in the mornings, usually up until midday except when mosquitoes were very scarce and collections had to be continued until mid-afternoon. All mosquitoes were collected by hand using an aspirator. Live mosquitoes were identified morphologically as belonging to either the *Anopheles gambiae *complex or the *An. funestus *subgroup and used in insecticide susceptibility tests. They were subsequently stored on dry silica gel for laboratory processing.

### WHO insecticide susceptibility tests

The WHO protocol [6] was used for testing susceptibility to the four classes of insecticides approved for vector control (pyrethroids, carbamates, organophosphates and organochlorines) except that age and physiological status of the mosquitoes was unknown. Treated test papers with the WHO diagnostic dosages were supplied by the WHO Collaborating Centre in Penang, Malaysia (Table 2). Cohorts of ~25 mosquitoes were exposed for 1 hour to the discriminating dose (except for fenitrothion which requires a 2-hour exposure [7]) and held for 24 hours before final mortality was recorded. Control mosquitoes were handled in the same way but not exposed to insecticides and were added to the following day's collections to maximise the numbers tested, particularly when population density was low. Treated papers used in the field were tested in the laboratory using known susceptible mosquitoes to check for efficacy. The dead and alive mosquitoes were stored separately for species identification. WHO criteria for susceptibility are: 98-100% mortality - susceptible; 80 - 97% mortality - resistance suspected and more investigations required; 0 - 79% mortality - resistance confirmed.

### Laboratory analysis

Mosquitoes were processed for species identification using the standard protocols for the *An. gambiae *complex [8,9] and the *An. funestus *group [10]. DNA was sourced from legs and wings of individual mosquitoes, either directly by grinding in buffer or using the Collins *et al*. extraction method [11]. *Plasmodium falciparum *parasite detection was carried out on dissected heads and thoraces of wild females using the enzyme-linked immunosorbent assay (ELISA) of Wirtz *et al*. [12]. The target site *kdr *resistance mutations were assayed in mosquitoes exposed to pyrethroids (from the same DNA samples used for species identification) using the Taqman assay [13] for both East and West African mutations.

## Results

The collection of indoor resting mosquitoes is very haphazard. Some houses in a village proved to be very productive with lots of mosquitoes present while other houses were not. Where treated bed nets in good condition were present, no mosquitoes were found in the bedrooms but on some occasions many mosquitoes were found resting inside the treated nets as well as in the adjacent living rooms, behind chairs or under tables, obviously avoiding the bedrooms due to the repellent effect of the treated bed nets [14].

Of the 1704 specimens of the *An. gambiae *complex (76.4% of 2229) that were successfully identified, all but one was *An. gambiae s.s*. (a single *An. arabiensis *was identified from Ahafo). A subsample of these (n = 862) was further characterised for the molecular forms and most were identified as the S molecular form (93.4%) (Table 1). The molecular M form was found in low numbers at Ahafo (6.8% of 220) and Tarkwa (8.3% of 459) while M/S hybrids (n = 4) were found only at Tarkwa. *Anopheles funestus s.s*. was the only member of the group identified from all localities (Table 1) except for a single specimen of *An. leesoni *being found at Tarkwa.

**Table 1:** Species identification and infection rates with *Plasmodium falciparum *circumsporozoite protein.

	*funestus*	*arabiensis*	*gambiae s.s*.	S form	M form	M/S hybrids
**Obuasi**						

No. Identified	181	0	633	160	0	0

% infected	1.81* (4/221)	-	-	4.9* (13/267)	-	-

**Tarkwa**						

No. Identified	78	0	475	417	38	4

% infected	3.8 (3/78)	-	4.7 (17/365)	4.5 (15/333)	6.25 (2/32)	0

**Ahafo**						

No. Identified	34	1	461	205	15	0

% infected	2.9 (1/34)	-	8.3 (19/229)	8.6 (16/187)	15.4 (2/13)	-

**Akyem**						

No. Identified	100	0	134	23	0	0

% infected	8.06 (5/62)	-	5.03 (8/159)	-	-	-

*These infectivity rates for Obuasi are taken from Coetzee *et al*. [2] for comparison as no ELISAs were carried out in 2010.

**Table 2:** Results of exposure of wild *Anopheles gambiae *S form adults to insecticide treated papers using the WHO susceptibility test.


**Locality**	**Insecticide class**	**Treated papers**	**Length of exposure**	**N**	**% Mortality 24 h post-exposure**

Obuasi	Pyrethroids	0.05% Deltamethrin	1 hour	64	89.1
		0.05% Lambda-cyhalothrin		60	76.7
		0.15% Cyfluthrin		62	66.1
		0.75% Permethrin		65	38.5

	Carbamates	0.1% Bendiocarb	1 hour	61	93.4
		0.1% Propoxur		59	89.8

	Organophosphates	1.0% Fenitrothion	2 hours	60	96.7

	Organochlorines	4.0% DDT	1 hour	60	31.7

Tarkwa	Pyrethroids	0.05% Deltamethrin	1 hour	152	56.6

	Carbamates	0.1% Bendiocarb	1 hour	113	74.3

	Organophosphates	5.0% Malathion	1 hour	210	98.6

	Organochlorines	4.0% DDT	1 hour	39	5.4
		4.0% Dieldrin		14	0

Akyem	Pyrethroids	0.05% Deltamethrin	1 hour	173	75.1

	Carbamates	0.1% Bendiocarb	1 hour	56	37.5

	Organophosphates	5.0% Malathion	1 hour	51	98.0

	Organochlorines	4.0% DDT	1 hour	53	5.7
		4.0% Dieldrin		56	0

Ahafo	Pyrethroids	0.05% Deltamethrin	1 hour	186	45.1
		0.05% Lambda-cyhalothrin		27	19.0
		0.75% Permethrin		41	15.0

	Carbamates	0.1% Bendiocarb	1 hour	178	77.6

	Organophosphates	5.0% Malathion	1 hour	171	95.4
		1.0% Fenitrothion	2 hours	29	93.1

	Organochlorines	4.0% DDT	1 hour	100	3.1
		4.0% Dieldrin		89	9.2

Each day's exposures were run with at least 20 control mosquitoes. All controls showed < 5% mortality negating the need for the use of Abbott's formula [6].

The results of the susceptibility tests for 2229 *An. gambiae s.l*. are given in Table 2. In all cases the control mortality was less than 5%, therefore not requiring adjustment with Abbott's formula [6]. Only the organophosphates showed good efficacy across all localities. High frequencies of resistance were recorded to pyrethroids, DDT and carbamates.

All the *An. funestus *tested were 100% susceptible to deltamethrin (n = 166) and malathion (n = 251) at all four localities. Samples from three localities were tested on bendiocarb and resistance was detected only at Obuasi (71.4% mortality, n = 56 [2]) but sample sizes were small at the other two sites (Tarkwa 53, Ahafo 3). DDT resistance was recorded at Obuasi (60.9% mortality, n = 23 [2]) but insufficient samples for testing this insecticide were collected at the other sites.

The *kdr *results are given in Table 3. As expected for *An. gambiae *S form in this area of West Africa, *kdr *frequencies of the West African mutation, Leucine to Phenylalanine (L1014F), were high. The East African mutation, Leucine to Serine (L1014S), was not found in any of the specimens. Only five specimens of *An. gambiae *M form were available (from Tarkwa) for *kdr *analysis and one was heterozygous for L1014F. The single M/S hybrid that survived exposure to deltamethrin was homozygous RR.

**Table 3:** Knockdown resistance (*Kdr*) mutations (L1014F) in wild *Anopheles gambiae*, exposed to pyrethroid insecticide treated papers, from four localities in Ghana.


**Locality**	**Molecular form**	**Resistant** **Phenotype**	**RR**	**RS**	**SS**	**% *kdr *** **frequency**

Obuasi	S	Resistant	11	1	0	95.8

		Susceptible	12	31	8	53.9

Ahafo	S	Resistant	20	0	0	100

Akyem	S	Resistant	17	18	3	68.4

		Susceptible	14	7	6	64.8

Tarkwa	S	Resistant	56	1	0	99.1

		Susceptible	10	0	0	100

Genotypes RR = homozygous resistant, RS = heterozygous resistant/susceptible and SS = homozygous susceptible.

The *Plasmodium falciparum *infection rates are given in Table 1 with Obuasi rates taken from the published literature [2]. Since only a small number of the Akyem *An. gambiae s.s*. were further identified to S form, the infection rate is given for "*gambiae s.s*.". The highest infection rates were recorded at Ahafo with over 8% for the S form and for the pooled *"gambiae s.s."*. The rate of 15% for the M form is to be treated with caution given the small sample size (n = 13). Infection rates for *An. funestus *were much lower than *An. gambiae *at three of the four sites but higher for *An. funestus *at Akyem.

## Discussion

The most common species encountered was *An. gambiae *S form, with very few M form and only one *An. arabiensis *identified. Since the surveys were carried out on an *ad hoc *basis and usually timed for the middle of the rainy season, it is possible that the species composition varies depending on the time of the year. *Anopheles funestus *appeared to play a secondary role as a vector except at Akyem. It has a scattered distribution and was found only at sites where suitable swampy breeding habitats were abundant.

All insecticide exposures were carried out on wild female mosquitoes of unknown age and physiological status. Since knowing the age of the samples is useful for early detection of developing resistance, when resistance is already established and as high as in the present study, the age effect [15] becomes less important. Furthermore, while age effect has been demonstrated for DDT resistance in *An. gambiae *[15] and permethrin resistance in *An. funestus *[16,17], it is not known whether the same applies to the organophosphates and carbamates for these species.

Mosquitoes of all physiological stages were collected, from unfed, to fully fed and gravid. The status of each individual was not recorded which means that susceptibility results give an overall view of resistance at each locality and cannot be broken down into physiological groups for comparison. Blood feeding is known to enhance the ability of female mosquitoes to survive exposure to some insecticides in some vector species (e.g. *An. funestus *[18]) but this may not be true for others (e.g. *An. gambiae *[19] except that this study did not expose freshly blood fed females but rather females that had been given a blood meal 6 days prior to exposure). The direct testing of wild females, as opposed to F-1 adults, is therefore pertinent for operational control even if it leads to an over-estimation of true (genetic) resistance in a population. Choice of insecticide for control interventions must be based on the number of mosquitoes that can survive exposure to any given insecticide regardless of age or physiological status.

Where very low mortality rates were recorded in the first round of testing, no further tests were conducted on that insecticide resulting in small sample sizes for some of the assays. In many cases mosquitoes were hard to come by and samples were used judiciously to provide maximum data on possible candidate insecticides for use in vector control by the various mines. As stated above, the surveys were conducted for only a short period at each locality, at times of the year that may not have been conducive to sampling other vector species such as *An. gambiae *M form, *An. arabiensis *and *An. funestus*. It is possible that these species can be found in greater numbers at other times of the year and that they may have different insecticide resistance profiles.

In general, the data confirm that resistance to one member of a class of insecticides is good evidence that resistance to other chemicals in the same class will occur. This is certainly the case with the pyrethroids tested here and is cause for concern. This insecticide group contains the only insecticides approved for use on bed nets. This valuable vector control strategy, where the person to be protected also serves as the bait to lure the mosquito into absorbing a lethal dose of insecticide, thus achieving the 'mass killing' effect [20], can no longer be relied upon. The diminishing effectiveness of this control tool comes at a time when international support for malaria control is significantly improving bed net coverage in Africa. During the study conducted at Ahafo in 2008 it was commonplace to find *An. gambiae *mosquitoes fully blood fed and resting inside long-lasting nets during the day. These observations were not quantified, but the study by N'Guessan *et al*. [21] in neighbouring Benin provides data for this phenomenon and highlights a very real problem facing control programmes in this region of West Africa.

It is probable that the increasing use of ITNs in the area is impacting on the levels of resistance to pyrethroids and DDT. In 2000, Kristan *et al*. [22] collected *An. gambiae s.s*. (no molecular identifications done) from the Tarkwa area and reported 100% 24-hr mortality on deltamethrin, 99.2% on permethrin and 94% after an 80 min exposure on DDT. This is in sharp contrast to the results presented here where 24-hr mortality was 56.6% on deltamethrin and only 5.4% on DDT from samples collected seven years later. It is obvious that this population has undergone considerable selection pressure and that resistance has increased exponentially over a very short period. On the other hand, the susceptibility results published for Obuasi in 2006 [2] show much lower mortality rates than the 2010 results given here for pyrethroids and carbamates. This may be due to the 2010 collections coming from rural villages outside of the mining area whereas the 2006 results were based on mosquitoes collected inside Obuasi town (before IRS was implemented) where inhabitants commonly use treated bed nets, mosquito coils and aerosols thereby increasing the selection pressure for resistance. The very low levels of mortality on 0.75% permethrin at Obuasi (38.5%) and Ahafo (15%) are of grave concern for the continued use of permethrin-treated bed nets.

Yawson *et al*. [23] report high frequencies of the Leu-Phe *kdr *mutation in Ghana populations with 100% recorded from Kumasi which is just north of Obuasi and in close proximity to Ahafo and Akyem (Figure 1). In the present study, of 63 specimens from Obuasi processed for *kdr*, 61.9% carried the Leu-Phe mutation, with 91.7% of the deltamethrin survivors being homozygous *kdr*. In Ahafo there was 100% *kdr *in 20 surviving individuals and in Akyem the overall frequency of *kdr *was 66.9%. Other recent studies in the vicinity of Accra also showed high *kdr *frequencies [24-26]. The almost 100% *kdr *mutations recorded from Tarkwa, including those samples that were susceptible to deltamethrin, indicates that a metabolic mechanism must play a role in conferring resistance to pyrethroids and that *kdr *on its own is not sufficient to confer resistance to an individual mosquito.

Working in northern Ghana near Navrongo, Anto *et al*. [27] reported worrying trends in *An. gambiae *and *An. funestus *survival on four pyrethroids and DDT. Despite their assertion that the vectors were all susceptible to these insecticides, in some instances survival was as high as 20%. In the limited number of specimens that were processed in the laboratory, no *kdr *mutations were found and all *An. gambiae *specimens were identified as the M molecular form. This suggests that a metabolic mechanism is responsible for the resistance observed in this region of Ghana as well.

## Conclusions

The data presented here clearly indicate that insecticide resistance is widespread and often at very high frequencies, usually sufficiently high to preclude the use of several of the few insecticides approved by WHO for malaria control. Resistance management strategies are therefore critical for vector control programmes in this region.

## Competing interests

The authors declare that they have no competing interests.

## Authors' contributions

RHH and MC were responsible for the design of the projects, data analysis and drafting the manuscript. GF, SK, JS-O and RV were responsible for the field logistics, data collection and drafting of the manuscript. MK, KSC and LLK were responsible for the laboratory data collection and analysis and drafting of the manuscript. All authors read and approved the final version of the manuscript.
